# The Severity of Depression, Anxiety, and Stress: Recommendations From Joint Work of Research Center and Psychology Clinics in COVID-19 Pandemic

**DOI:** 10.3389/fpsyt.2022.839542

**Published:** 2022-06-20

**Authors:** Hira Shahid, Muhammad Abul Hasan, Osama Ejaz, Hashim Raza Khan, Muhammad Idrees, Mishal Ashraf, Sobia Aftab, Saad Ahmed Qazi

**Affiliations:** ^1^Neurocomputation Laboratory, National Center of Artificial Intelligence, Karachi, Pakistan; ^2^Department of Biomedical Engineering, NED University of Engineering and Technology, Karachi, Pakistan; ^3^Department of Electronics Engineering, NED University of Engineering and Technology, Karachi, Pakistan; ^4^Al'Shakoor Mental Health Clinic, Al'Shakoor Foundation, Karachi, Pakistan; ^5^Rehabilitation Centre for Drug Addicts, New Horizon Care Centre (NHCC), Karachi, Pakistan; ^6^Institute of Clinical Psychology, University of Karachi, Karachi, Pakistan; ^7^Department of Electrical Engineering, NED University of Engineering and Technology, Karachi, Pakistan

**Keywords:** depression, anxiety, stress, mental health, COVID-19

## Abstract

The COVID-19 pandemic has significantly affected the psychological stability of general population of Pakistan. However, research on the severity of COVID-19 induced depression, anxiety, and stress (DAS) in Pakistan is scarce. This paper thereby investigates the severity of COVID-19 induced DAS based on demographic, socioeconomic, and personal feeling variables by modeling DAS. Snowball sampling strategy was adopted to conduct online survey from July 03, 2021 to July 09, 2021. Out of 2,442, 2,069 responses from Karachi were included. Descriptive and inferential statistics (binary and multinomial logistic regression analysis) were performed using SPSS V21 (IBM, 2013) to identify significant determinants and their association with DAS severity. The result of this study indicates 27.8, 21.7, and 18.3% respondents suffer from severe and extremely severe states of depression, anxiety, and stress, respectively. Binary logistic regression revealed that age is a significant determinant with odds of having 4.72 (95% CI = 1.86–11.97) and 5.86 (95% CI = 2.26–15.2) times greater depression, and stress for respondents aged 19–26 years. Moreover, gender-based difference is also observed with females 1.34 (95% CI = 1.08–1.68) and 1.75 (95% CI = 1.40–2.20) times more likely to exhibit anxiety and stress than males. Furthermore, marital status is a significant determinant of depression with odds of having depression is 0.67 (95% CI = 0.48–0.93) times greater for married population. Multinomial logistic regression revealed that those who believe COVID-19 pandemic has affected them mentally, fear new COVID-19 cases and deaths, depressed due to imposition of lockdown, believe they will not survive COVID-19 infection, and spend more time on social media gathering COVID-19 updates suffer from extremely severe state of depression (OR mental-effect-of-pandemic = 3.70, OR new-COVID-19-cases-and-deaths = 2.20, OR imposition-of-lockdown = 17.77, OR survival-probability = 8.17, OR time-on-social-media = 9.01), anxiety (OR mental-effect-of-pandemic = 4.78, OR new-COVID-19-cases-and-deaths = 3.52, OR imposition-of-lockdown = 5.06, OR survival-probability = 8.86, OR time-on-social-media = 5.12) and stress (OR mental-effect-of-pandemic = 6.07, OR imposition-of-lockdown = 11.38, OR survival-probability = 15.66, OR time-on-social-media = 4.39). Information regarding DAS severity will serve as a platform for research centers and psychological clinics, to work collectively and provide technology-based treatment to reduce the burden on the limited number of psychologist and psychotherapist.

## Introduction

The SARS-CoV-2 (COVID-19) outbreak was first reported in Wuhan, China, at the end of December 2019 and has swiftly disseminated across the globe in early March 2020 (1–5). The novel COVID-19 virus was first reported in Karachi, Pakistan, in February 2020 as confirmed by the Pakistan Ministry of Health (6). Following which, community spread begins making Karachi the epicenter of COVID-19 virus (7). The COVID-19 confirmed cases have reached to 250 million by far with around 5 million deaths (8). In Pakistan alone, the count of individuals infected from COVID-19 is approaching 1.3 million with ~30,000 deaths (9).

In an attempt to curb the spread of COVID-19 infection, extreme preventive measures such as lockdown, social distancing, social isolation, and quarantine were adopted which have induced fear in people at individual, societal, and international levels (1, 10, 11). The prevalence of this infectious diseases not only involves risks of lives but also threatens the psychological wellbeing of masses as documented in Asian Journal of Psychiatry (6, 12). The psychological symptoms of depression, anxiety, and stress associated with such deadly infectious diseases are due to precarious situation of employment, social capital, and sociocultural factors such as economic burden, family and society support, and low quality education (1, 8, 9, 13–18). Moreover, the increasing popularization and interaction with electronic and social media regarding COVID-19 updates have further exacerbated the mental wellbeing of general population as indicated in former studies (19, 20).

The COVID-19 pandemic incurred massive mental health issues worldwide. It has an intense effect on mental health, reporting the prevalence of depression, anxiety, and stress at 47, 47, and 44%, respectively, in lower-middle-income countries (21). It is also observed that mental health problems are significantly associated with suicide (22, 23). In addition, sleep problem has also been reported high in a nationwide sample of Bangladesh, i.e., 30.4, 13.1, and 2.8% of participants reported sub-threshold, moderate, and severe forms of insomnia, respectively (24). During the pandemic time, fear of COVID-19 was also a significant factor of mental disorders in Bangladesh Students (25). Higher psychological distress (41.8%), poor sleep quality (57.1%), increased anxiety (37.19%), along with higher cutoff ratings for psychopathological symptomatology (31.38%) and PTSD (29.5%) have been reported in the Italian population (26–28). A deteriorated mental health status with increased prevalence of depression and anxiety by 19 and 14% in Hong Kong, 20 and 22.8% in Ireland, and 23.6 and 45.1% in Turkey has been found in former studies (29–31). Higher odds of anxiety and depression symptomatology were found in the American population as a consequence of the COVID-19 outburst (32). More than half of the respondents reported a profound psychological impact during COVID-19 outbreak in china (1). A previous study reported fear in general public for contacting virus during influenza outburst (33). Likewise, lower rate of quality education and awareness regarding infectious pandemic is also found associated with mental suffering as indicated in a prior study (18). Economic recession with a major cut in salaries and unemployment, and negative emotions experienced by people are intensified during isolation (13–16). Socio-cultural factors such as economic burden, family support, and social support areas are also deemed essential to influence mental health (8). A strong influence of social capital has been reported in a study conducted in china during COVID-19 (8).

The psychological impacts of the COVID-19 in Pakistan could be catastrophic with only 0.48 psychologist and psychotherapist per 1,000 population (34). Prior to the pandemic, literature documenting pathological stress among working Pakistani adults also showed increased risk of anxiety and depression-related symptoms if stress was not appropriately addressed. Furthermore, the prevalence of extreme depression, anxiety, and stress is relatively high in those under 30 (35–40). The COVID-19 pandemic has the potential to exacerbate a lot of these conditions, which can lead to worst mental health conditions.

Given the large span of COVID-19 outbreak along with the social and economic instabilities that accompanied it, the psychological problems associated with it will persist even after the outbreak is over. The prevalence of COVID-19 induced depression, anxiety, and stress is available for the general population of Pakistan (36–41), but information related to its level of severity is not available. Keeping in view the limited number of psychologist and psychotherapist in Pakistani setting, the severity level of COVID-19 induced depression, anxiety, and stress demands serious attention. This study conducted in collaboration with clinical psychologist thereby aims to fill the research gap by investigating the prevalence of COVID-19 induced depression, anxiety, and stress along with the magnitude of severity based on demographic, socioeconomic, and personal feeling variable in Karachi, Pakistan, by modeling depression, anxiety, and stress. We hypothesized that the increase in the prevalence of depression, anxiety, and stress will result in an increased severity level of COVID-19 induced depression, anxiety, and stress. In addition to the clinical psychologist, this study also has academic institutes on board to focus on the distribution of the general population of Pakistan depending on the severity levels of depression, anxiety, and stress. The collaboration with academic institute will facilitate data collection from young population which constitutes large proportion of Pakistan's general population. Moreover, the collaboration with healthcare sector and research institutes will also allow appropriate management of each stage of DAS without burdening the limited number of psychologist and psychotherapist available in Pakistani setting by introducing technology in healthcare sectors for assessment and treatment of mental health issues.

## Methods

### Population and Sample Size

A total of 2,442 respondents participated in the study from all over Pakistan. Out of these, 373 responses from provinces and states other than Karachi were excluded as these were from non-urban areas. This small number of responses was excluded to avoid biasness and ensures homogeneous sample population. Eventually, we included 2,069 respondents who had completed and submitted the questionnaires from Karachi—a city with then reported 203,716 COVID-19 cases (42).

### Ethical Considerations

Consent was obtained from Nadirshaw Eduljee Dinshaw (NED) University of Engineering and Technology's ethical review committee in Karachi, Pakistan. This questionnaire was developed using Google forms. The survey comprises of a contextual overview, inclusion criteria, directions on answering questions, informed consent, and details of mental healthcare providers as well as principal investigator for reference. There was no anticipated psychological risk associated with participation in the study. All of the participants voluntarily agreed and provided their informed consent to participate in the study by clicking on the “I agree to participate” option provided in the online form. No electronic signature of the participants was obtained to avoid misuse and ensure participant comfort. To ensure privacy, anonymity, and confidentiality, no personal information about the participants was obtained. Demographic information of state and area was required and appeared prior to the questionnaire. Since children under 18 lack intellectual capacity as identified from previous research (43), participants below 18 years of age were asked to fill questionnaires in presence of their parent or guardian to avoid misinterpretation of the questions. This was made certain with a checkbox for under 18 respondents only.

### Inclusion/Exclusion Criteria and Data Collection Procedures

Our participants came predominately from Karachi, the most populous metropolitan city of Pakistan and the country's financial hub with highest COVID-19 cases (more than 250 thousand) (44–46). The nationals of Pakistan living in different countries were excluded from this study to analyze the severity and intensity of psychological distress during COVID-19 outbreak. Moreover, authors who have designed the survey were also excluded from the study to control biasing. Furthermore, a screening question regarding whether the respondent has consulted a psychologist in the past 2 years has enabled exclusion of the respondents having a history of psychiatric disorders. This was done to maintain homogeneity in the sample and to control the potential impact of prior history of psychological problems on current psychological problems.

### Recruitment Strategy

A cross-sectional survey design with a snowball sampling strategy was employed to recruit general public of Pakistan. Data were collected from July 03, 2021 to July 09, 2021. In adherence to social distancing practices as recommended by the Government of Pakistan, potential respondents were electronically invited by the investigators. A multilingual survey was designed that comprises of standard English language accompanied with its local Urdu translation in order to overcome the language barrier and facilitate the residents of Pakistan having limited English proficiency (47). The purpose to develop a multilingual survey was to eliminate the likelihood of misinterpretation of the survey questions which is quite frequent when the respondents have a limited English proficiency (48). The general public from all the provinces of Pakistan were invited to take this short assessment of up to 8 mins to help in identification of the factors that are primary contributors of stress, anxiety, and depression during the pandemic. This assessment could only be taken once. The response for each participant was restricted to a single response. The confidential assessment involves minimum risk and does not promise any reward of your participation and neither guarantee any benefit from this study. The survey was disseminated *via* WhatsApp Inc., Facebook Inc., and other social media platforms that includes LinkedIn and Twitter. The participants were also encouraged to circulate the survey to their contacts.

### Survey Development

Our questionnaire was divided into two parts: (1) Generalized Questionnaire and (2) Mental health Questionnaire.The generalized questionnaire consisted of questions related to: (1) demographic; (2) socioeconomic status; and (3) personal feeling during COVID-19 pandemic to identify the factors responsible for depression, anxiety, and stress specifically related to the impact of COVID-19 which were identified from literature (49–55).

Demographic variable of generalized questionnaire collected data regarding gender, age, education, and marital status. In the demographic variable, the factor of gender includes male and female. The factor of age is divided into 15–18 years, 19–26 years, 27–40 years, 41–60 years, and above 60 years of age. The factor of education includes bachelor/diploma, F.Sc./intermediate, illiterate, master, matric, and PhD. The factor of marital status is categorized into single, married, divorced, and widow.

Socioeconomic variable of generalized questionnaire included information of the respondents for variation in earning during COVID-19, household size, profession, and engagement in indoor activities during COVID-19 pandemic. The factor of variation in earning during COVID-19, in socioeconomic variable, includes decrease in earning, increase in earning, no change in earning, loss of job, and not applicable (mostly for students). The factor of household size collects information regarding the number of dependents that includes 0–4 dependents, 5–7 dependents, 8–10 dependents, and more than 10 dependents. The profession factor is categorized into corporate owner, government employee, housewife, private sector employee, retired, self-employed, shopkeeper, student, and those who are unemployed. The factor of engagement in indoor activities gathers information of participant involvement in indoor activities, participants not involved in indoor activities, and those who are somewhat involved in indoor activities.

Variable of personal feeling explicitly describes the thoughts of the responders regarding the pandemic and is not standardized. The parameters of personal feelings in the generalized questionnaire included the survival chances if infected with COVID-19 virus, psychological impact during exposure to a public health crisis, time spent on TV and social media collecting COVID-19 updates, the trend of new cases and death, and effect on mood of the respondents during lockdown. The factor of survival chances if infected with COVID-19 virus is categorized into those who believe that they will get well soon, those who believe that they will not survive COVID-19 infection, and those who are not sure. The factor of psychological impact during exposure to a public health crisis collects information regarding those who believe that COVID-19 pandemic has affected them mentally, those who believe that pandemic have not affected them mentally, and those who are not sure of the effect of pandemic on their mental health. The factor of time spent on TV and social media collecting COVID-19 updates is categorized into <1 h spent on social media, 1–3 h spent on social media, 4–6 h spent on social media, and more than 6 h spent on social media. Anxious, depressed, fear, upset, worried, and no effect are the subcategories for the factors of effect of lockdown as well as new cases and death.

For assessment of mental health, an internationally accepted and widely used Depression, Anxiety and Stress Scale (DASS-21) was employed as mental health questionnaire in this study to determine the severity of COVID-19 induced depression, anxiety, and stress (49–52, 56). It is a reliable tool for evaluating emotional states of depression, anxiety, and stress. Formerly, it has also been used for SARS research (57, 58). The 21 questions in the DASS-21 questionnaire are divided into seven questions for each emotional states, including depression, anxiety, and stress (57, 58). More information about survey makeup and clinical outcomes can be found in previous study (58). Briefly, each item score is on a scale of four-point ranging from 0 to 3. The anxiety subscale comprises of question 2, 4, 7, 9, 15, 19, and 20. The total anxiety subscale score was separated into five categories: normal (0–6), mild anxiety (7–9), moderate anxiety (10–14), severe anxiety (15–19), and extremely severe anxiety (20–42). The depression subscale comprises of question 3, 5, 10, 13, 16, 17, and 21. The total depression subscale score was separated into five categories: normal (0–9), mild depression (10–12), moderate depression (13–20), severe depression (21–27), and extremely severe depression (28–42), whereas question 1, 6, 8, 11, 12, 14, and 18 constitutes stress subscale with categorization into normal (0–10), mild stress (11–18), moderate stress (19–26), severe stress (27–34), and extremely severe stress (35–42).

### Statistical Analysis

Investigators stored data in a Microsoft Excel template (Microsoft, 2019) and exported the results to SPSS V21 (IBM, 2013) for statistical analysis. We used frequencies and percentages along with means and standard deviations to analyze the distribution of respondents among normal, mild, moderate, severe, and extremely severe state of DASS subscale.

We used binary logistic regression model to identify the strong determinants of depression, anxiety, and stress. The model used depression, anxiety, and stress as dependent variables and demographic, socioeconomic, and personal feeling factors as independent variables. For this purpose, DASS subscale results were transformed into “dummy variables,” represented with non-symptomatic and symptomatic. Respondents who were normal on DASS scale were referred to as non-symptomatic. Symptomatic, in this instance, referred to anyone who scored “mild,” “moderate,” “severe,” and “extremely severe” on the DASS. We then created three binary logistic regression models for each subscale. Wald coefficient was obtained to determine strength of significant determinants. *p* < 0.05 was set as significance level. Beta and confidence interval were obtained. A Homer–Lemeshow test was conducted to determine the goodness of fit. The 2 log likelihood ratio was used to determine the significance of the logistic models, while a Cox-Snell R2 and a Nagelkerke 2 were used to determine the value of each of the determinant to the outcome of interest.

We also performed multinomial logistic regression analysis and created three distinct models each for depression, anxiety, and stress to determine the association of level of severity of DASS subscales with the personal feeling variable identified significant from binary logistic regression. For each of the three models, only psychological determinants identified as significant with the binary regression model were used as independent variables. Only the dependent variable differs between the three models with depression being dependent variable in model 1, anxiety in model 2, and stress in model 3. *p* < 0.05 was set as significance level. Crude odds ratio and confidence interval were obtained. The goodness of fit was determined *via* Pearson's chi-square, and extreme variables were determined through deviance residuals. The Cox-Snell R2 and a Nagelkerke 2 were used to determine the value of each of the predictors to the outcome of interest, while Mc Fadden was used to determine the effect size.

## Results

Table 1 presents the distribution of the respondents in demographic, socioeconomic, and psychological variables. The distribution of the demographic variable reveals that the majority of the respondents were male (56.1%), aged 19–26 (49.3%), single (59.8%), and well-educated with or enrolled in bachelor's degree (50.5%). The socioeconomic distribution reflects that a large amount of the study population was private sector employee (32.9%), 0–4 dependents (66.6%), no change in earning (41.3%) or decrease earning (37.2%), and engaged in indoor activities (39.2%). Moreover, the distribution of respondents based on psychological variable indicates that the greater proportion of the study population was mentally affected with COVID-19 pandemic (49.3%), worried with the increasing new COVID-19 cases and deaths (32.7%), depressed as a consequence of lockdown (22.9%), believe that they will get well soon (72.2%), and spent <1 h on social media (75.9%).

**Table 1:** Descriptive statistics of the study population.

**Variables**		**Subcategories**	**N**	**Percentage**
Demographic	Gender	Female	908	43.9%
		Male	1,161	56.1%
	Age	15 years−18 years	75	3.6%
		19 years−26 years	1,019	49.3%
		27 years−40 years	663	32.0%
		41 years−60 years	258	12.5%
		60+	54	2.6%
	Marital status	Divorced	19	0.9%
		Married	800	38.7%
		Single	1,237	59.8%
		Widow	13	0.6%
	Level of education	Bachelor/Diploma	1,046	50.5%
		F.Sc. /Intermediate	261	12.6%
		Illiterate	4	0.2%
		Master	659	31.9%
		Matric	26	1.2%
		PhD	73	3.5%
Socioeconomic	Indoor activities	No	590	28.5%
		Sometimes	668	32.3%
		Yes	811	39.2%
	Earning comparison	Decreased	770	37.2%
		Increased	24	1.2%
		NA	270	13.0%
		No change	854	41.3%
		No earning/Lost my job	151	7.3%
	Dependents	0–4	1,378	66.6%
		5 to 7	553	26.7%
		8 to 10	107	5.2%
		More than10	31	1.5%
	Profession	Corporate owner	24	1.2%
		Government employee	263	12.7%
		Housewife	148	7.2%
		Private sector employee	681	32.9%
		Retired	31	1.5%
		Self-employed / Freelancer	122	5.9%
		Shopkeeper	18	0.9%
		Student	666	32.2%
		Unemployed	116	5.6%
Person feeling	Effect of lockdown	Anxious	436	21.1%
		Depressed	473	22.9%
		Fear	55	2.7%
		No effect	446	21.6%
		Upset	264	12.8%
		Worried	395	19.1%
	Time on social media	1 h – 3 h	368	17.8%
		4 h – 6 h	77	3.7%
		< an h	1,571	75.9%
		More than 6 h	53	2.6%
	New COVID-19 cases and deaths	Anxious	190	9.2%
		Depressed	346	16.7%
		Fear	196	9.5%
		None	158	7.6%
		Upset	503	24.3%
		Worried	676	32.7%
	Mental effect of pandemic	May be	474	22.9%
		No	574	27.7%
		Yes	1,021	49.3%
	Survival chances	I will get well soon	1,494	72.2%
		I will not survive	35	1.7%
		Not sure	540	26.1%

Table 2 presents distribution of respondent within each of the five categories (normal, mild, moderate, severe, and extremely severe) of DASS subscales measured with 21-item DASS scale. Out of 2,069 respondents, 810 (39.1%), 1,074 (51.9%), and 965 (46.6%) were found to be asymptomatic of depression, anxiety, and stress, whereas 1,259 (60.9%), 995 (48.1%), and 1,104 (53.4%) were identified to be symptomatic (mild, moderate, severe, and extremely severe) with 29.8%, 21.7%, and 18.3% severe and extremely severe cases for depression, anxiety, and stress.

**Table 2:** Descriptive statistics of depression, anxiety, and stress.

**Sub levels**	**Depression**		**Anxiety**		**Stress**	
	**N**	**%**	**N**	**%**	**N**	**%**
Normal	810	39.1	1,074	51.9	965	46.6
Mild	247	11.9	186	9	437	21.1
Moderate	396	19.1	360	17.4	289	14
Severe	233	11.3	133	6.4	205	9.9
Extremely severe	383	18.5	316	15.3	173	8.4
Total	2,069	100	2,069	100	2,069	100

Table 3 presents binary logistic regression to identify the strong determinants from generalized variables for depression, anxiety, and stress. The demographic variable “marital status” (Wald = 6.66) is found to be a strong determinant of depression with the odds of 0.67 for married respondents as opposed to unmarried respondents. Furthermore, factor “gender” is found to be a strong determinant of anxiety (Wald = 7.03) and stress (Wald = 24.20) with females being 1.34 and 1.75 times more likely to exhibit anxiety and stress, respectively, than males. Moreover, “age,” another demographic variable, is found to be a strong determinant of depression (Wald = 12.46) and stress (Wald = 16.52). Decreasing age was associated with an increased likelihood of exhibiting depression and stress. The respondents aged 19–26 years are most likely to suffer from depression (Wald = 10.69, OR = 4.72) and stress (Wald = 13.27, OR = 5.86) than those aged above 60 years.

**Table 3:** Determinants of depression, anxiety, and stress identified with binary logistic regression.

**Groups**	**Variables**	**Depression**					**Anxiety**					**Stress**				
		**Wald**	**OR**	***P*** **value**	***CI*** **lower**	**CI** **upper**	**Wald**	**OR**	***P*** **value**	***CI*** **lower**	***CI*** **upper**	**Wald**	**OR**	***P*** **value**	***CI*** **lower**	***CI*** **upper**
	**Constant**	4.70	0.53	0.45			0.43	0.31	0.16				**0.21**			
**Demographic**	**Gender** (Female)	0.87	1.12	0.34	0.88	1.42	7.03	1.34	**0.00**	1.08	1.68	24.20	1.75	**0.00**	1.40	2.20
	Reference: Male															
	**Age**	12.46		**0.01**			7.46		0.11			16.52		**0.00**		
	**Reference:60** **+**															
	15 to 18	7.93	4.94	**0.00**	1.62	15.03	2.70	2.60	0.10	0.83	8.16	9.36	5.67	**0.00**	1.86	17.26
	19 to 26	10.69	4.72	**0.00**	1.86	11.97	6.27	3.54	**0.01**	1.31	9.53	13.27	5.86	**0.00**	2.26	15.20
	27 to 40	6.41	3.14	**0.01**	1.29	7.62	5.51	3.13	**0.01**	1.20	8.14	8.52	3.89	**0.00**	1.56	9.68
	41 to 60	5.85	3.02	**0.01**	1.23	7.39	4.59	2.86	**0.03**	1.09	7.48	5.88	3.11	**0.01**	1.24	7.79
	**Marital status**	6.66		**0.00**			4.69		0.09			1.55		0.46		
	**Reference: Single**															
	Divorced /Widow	0.19	1.22	0.66	0.49	2.98	0.01	1.05	0.89	0.44	2.50	1.47	1.71	0.22	0.71	4.10
	Married	5.48	0.67	**0.01**	0.48	0.93	4.24	0.71	**0.03**	0.52	0.98	0.34	1.10	0.55	0.79	1.51
	**Level of education**	2.30		0.31			1.95		0.37			0.11		0.94		
	**Reference: PhD**															
	Illiterate	2.59	8.46	0.10	0.62	114.10	2.48	0.54	0.46	0.22	27.87	0.11	0.94	0.73	0.67	1.31
	Matric	2.20	7.09	0.13	0.53	93.85	2.99	0.79	0.37	0.27	33.05	0.01	0.98	0.91	0.76	1.27
	FSc./Intermediate	2.67	8.84	0.10	0.64	120.65	2.43	0.516	0.47	0.21	27.65	6.52	10.63	0.00	2.12	20.43
	Bachelor/Diploma	0.957	3.87	0.32	0.25	58.59	5.88	1.86	0.17	0.46	75.21	5.65	12.54	0.00	2.16	14.71
	Master	3.29	11.88	0.06	0.82	171.69	2.65	0.59	0.44	0.22	32.11	1.21	0.54	0.46	0.72	2.00
**Socio-economic**	**Indoor activities**	1.60		0.44			0.35		0.83			0.85		0.65		
	**Reference: Yes**															
	No	0.67	1.11	0.41	0.85	1.44	0.06	1.03	0.80	0.80	1.32	0.04	1.02	0.84	0.80	1.31
	Sometimes	0.26	0.93	0.60	0.73	1.19	0.35	1.07	0.55	0.85	1.34	0.80	1.11	0.36	0.88	1.40
	**Earning comparison**	20.92		**0.00**			7.87		0.09			1.07		0.89		
	**Reference: no earning**															
	Decrease	5.79	0.51	**0.01**	0.30	0.88	1.06	0.79	0.30	0.50	1.23	0.02	0.96	0.86	0.61	1.51
	Increase	0.00	1.00	0.99	0.31	3.19	0.49	1.45	0.48	0.51	4.07	0.47	1.43	0.49	0.51	3.96
	NA	12.60	0.35	**0.00**	0.19	0.62	2.59	0.66	0.10	0.40	1.09	0.11	0.91	0.74	0.55	1.52
	No Change	12.35	0.38	**0.00**	0.22	0.65	3.65	0.64	0.05	0.41	1.01	0.14	0.91	0.70	0.57	1.44
	**Dependents**	7.82		**0.00**			2.72		0.43			2.58		0.46		
	**Reference: more than 10**															
	0 to 4	1.21	0.60	0.27	0.24	1.47	0.22	1.22	0.63	0.53	2.81	0.01	1.05	0.89	0.46	2.39
	5 to 7	2.99	0.45	0.08	0.18	1.11	0.12	1.16	0.71	0.50	2.71	0.08	0.88	0.76	0.38	2.01
	8 to 10	1.09	0.59	0.29	0.22	1.58	0.11	0.85	0.73	0.33	2.15	0.05	0.89	0.81	0.36	2.23
	**Profession**	8.91		0.17			3.05		0.80			3.96		0.68		
	**Reference: Unemployed**															
	Corporate	0.00	1.01	0.96	0.54	1.86	0.77	1.28	0.37	0.73	2.25	0.06	0.92	0.79	0.52	1.63
	Government	1.01	1.42	0.31	0.71	2.84	1.72	1.52	0.19	0.81	2.86	0.00	0.99	0.99	0.52	1.89
	Housewife	0.10	0.91	0.75	0.52	1.59	0.44	1.18	0.50	0.72	1.95	0.00	1.00	0.98	0.60	1.66
	Private	1.85	2.36	0.17	0.68	8.18	0.10	0.80	0.75	0.21	3.00	0.50	1.56	0.47	0.45	5.34
	Retired	1.03	1.38	0.30	0.73	2.61	0.85	1.30	0.35	0.73	2.31	0.97	1.33	0.32	0.75	2.38
	Self-employed	0.41	1.20	0.51	0.68	2.10	1.16	1.31	0.28	0.80	2.15	0.49	1.19	0.48	0.72	1.98
	Shopkeeper	1.76	0.78	0.18	0.55	1.12	0.68	1.43	0.40	0.61	3.33	0.07	1.12	0.79	0.48	2.59
	Student	0.31	1.07	0.57	0.82	1.40	1.09	1.56	0.29	0.67	3.64	0.00	0.98	0.98	0.39	2.46
**Personal feeling**	**Effect of lockdown**	74.73		**0.00**			37.20		**0.00**			65.17		**0.00**		
	**Reference: no effect**															
	Fear	5.69	2.31	**0.01**	1.16	4.63	17.91	4.86	**0.00**	2.33	10.13	8.74	2.76	**0.00**	1.40	5.40
	Depressed	70.01	4.36	**0.00**	3.08	6.16	21.07	2.14	**0.00**	1.55	2.97	55.19	3.51	**0.00**	2.52	4.89
	Worried	22.94	2.22	**0.00**	1.60	3.07	3.99	1.39	**0.04**	1.00	1.94	11.99	1.77	**0.00**	1.28	2.45
	Anxious	37.49	2.74	**0.00**	1.98	3.78	14.74	1.87	**0.00**	1.36	2.58	31.90	2.50	**0.00**	1.82	3.44
	Upset	18.80	2.20	**0.00**	1.54	3.15	1.75	1.27	0.18	0.89	1.82	5.71	1.53	**0.01**	1.08	2.19
	**Time on social media**	17.60		**0.00**			15.66		**0.00**			10.82		**0.01**		
	**Reference:** **<** **1 h**															
	1h to 3 h	9.93	1.57	**0.00**	1.18	2.09	4.70	1.33	**0.03**	1.02	1.72	2.33	1.22	0.12	0.94	1.59
	4 h to 6 h	0.51	1.22	0.47	0.70	2.11	3.60	1.64	0.05	0.98	2.75	1.26	1.35	0.26	0.80	2.28
	More than 6 h	8.54	3.10	**0.00**	1.45	6.63	9.28	2.76	**0.00**	1.43	5.32	8.22	2.70	**0.00**	1.37	5.34
	**New COVID-19 cases and deaths**	27.32		**0.00**			23.99		**0.00**			21.00		**0.00**		
	**Reference: None**															
	Fear	12.64	2.74	**0.00**	1.57	4.79	11.99	2.61	**0.00**	1.51	4.50	8.34	2.18	**0.00**	1.28	3.71
	Depressed	11.92	2.37	**0.00**	1.45	3.89	11.92	2.38	**0.00**	1.45	3.90	3.86	1.60	**0.04**	1.00	2.58
	Worried	9.73	1.99	**0.00**	1.29	3.07	2.54	1.44	0.11	0.91	2.27	1.34	1.28	0.24	0.84	1.96
	Anxious	9.72	2.31	**0.00**	1.36	3.90	4.17	1.73	**0.04**	1.02	2.93	4.44	1.73	**0.03**	1.04	2.88
	Upset	1.58	1.32	0.20	0.85	2.05	2.55	1.45	0.11	0.91	2.29	0.00	1.00	0.98	0.65	1.54
	**Mental effect of pandemic**	57.60		**0.00**			71.59		**0.00**			49.53		**0.00**		
	**Reference: Yes**															
	No	55.92	0.36	**0.00**	0.27	0.47	67.67	0.33	**0.00**	0.25	0.43	41.89	0.42	**0.00**	0.33	0.55
	May be	18.22	0.56	**0.00**	0.43	0.73	22.79	0.55	**0.00**	0.43	0.70	24.97	0.53	**0.00**	0.41	0.68
	**Survival chance**	10.29		**0.00**			15.49		**0.00**			19.37		**0.00**		
	**Reference: not sure**															
	I will not survive	3.00	2.85	0.08	0.87	9.36	5.90	3.42	**0.01**	1.26	9.24	3.65	2.80	0.05	0.97	8.05
	I will get well soon	5.84	0.74	**0.01**	0.57	0.94	7.19	0.73	**0.00**	0.58	0.92	13.18	0.65	**0.00**	0.51	0.82

*p < 0.05 is represented with **bold***.

In the socioeconomic variable, the factors of “variation in earning” (Wald = 20.92) and “number of dependents” (Wald = 7.82) are found to be a strong determinants of depression only.

All the factors of personal feelings are found to be a strong determinant of depression, anxiety, and stress. The “mental effect of pandemic” is a significant factor for depression (Wald = 57.60), anxiety (Wald = 71.59), and stress (Wald = 49.53). The respondents who believe that the pandemic has not affected them mentally have reduced odds of having depression (OR = 0.36), anxiety (OR = 0.33), and stress (OR = 0.42). Moreover, the “effect of lockdown” is a significant factor for depression (Wald = 74.73), anxiety (Wald = 37.20), and stress (Wald = 65.17) with those who feel depressed were 4.36 and 3.51 times more likely to suffer from depression and stress, respectively, while those who fear lockdown implications are 4.86 times more likely to exhibit anxiety. Furthermore, factors such as “new COVID-19 cases and deaths,” “time on social media,” and “survival probability” are significant determinants of depression (Wald new COVID-19 cases and deaths = 27.32, Wald time on social media = 17.60, Wald survival chance = 10.29), anxiety (Wald new COVID-19 cases and deaths = 23.99, Wald time on social media = 15.66, Wald survival chance = 15.49), and stress (Wald new COVID-19 cases and deaths = 21.00, Wald time on social media = 10.82, Wald survival chance = 19.37). The respondents who fear new COVID-19 cases and deaths and those who spend more than 6 h on social media are 2.74 and 3.10 times more likely to suffer from depression, 2.61 and 2.76 times more likely to suffer from anxiety, and 2.18 and 2.70 times more likely to suffer from stress. Also, respondents who believe to have a greater survival chance are 0.74, 0.73, and 0.65 times less likely to suffer from depression, anxiety, and stress

Tables 4A–C presents multinomial logistic regression analysis for severity of depression, anxiety, and stress, respectively, as opposed to the respondents having normal DAS scores. For Table 4A, the factor “mental effect of pandemic” increases the likelihood of depression for the respondents who believe that the pandemic has affected them mentally and those who remains neutral regarding the mental effect of pandemic with mild (OR affected = 1.74), moderate (OR affected = 2.25, OR neutral = 1.51), severe (OR affected = 3.38), and extremely severe states (OR affected = 3.70, OR neutral = 1.55) of depression. In contrast to those who does not reported any effect of “new COVID-19 cases and deaths,” an increased likelihood of having depression is observed for the respondents who fear, depressed, worried, anxious, and upset with mild (OR fear = 2.48, OR worried = 2.69, OR anxious = 2.71), moderate (OR fear = 4.25, OR depressed = 2.63, OR worried= 3.15, OR anxious = 3.39, OR upset = 1.99), severe (OR fear = 2.34), and extremely severe (OR fear = 2.19, OR depressed = 2.20) states of depression. It is also observed that the likelihood of depression increases for the respondents who fear, depressed, worried, anxious, and upset from the “implication of lockdown” evident with mild (OR depressed = 2.37, OR anxious = 2.23, OR upset = 2.04), moderate (OR depressed = 3.74, OR worried = 2.43, OR anxious = 2.48, OR upset = 2.03), severe (OR fear = 2.59, OR depressed = 4.37, OR worried = 2.12, OR anxious = 2.61, OR upset = 2.68), and extremely severe (OR fear = 7.98, OR depressed = 17.77, OR worried = 4.89, OR anxious = 6.80, OR upset = 5.50) states of depression, as opposed to those with no affect. The increase in “time spent on social media” to gather COVID-19 updates increases the likelihood of severe (OR = 4.56) and extremely severe (OR = 9.01) state of depression. When compared with those who believe that they will “survive the COVID-19 infection,” the respondents who believe that they will have less or no survival probability are at higher likelihood of severe (OR not survive = 5.05) and extremely severe (OR not survive = 8.17, OR not sure = 1.76) state depression.

**Table 4A:** Association of level of severity of depression with the personal feeling variable identified with multinomial logistic regression.

**Depression**																
	**Mild**				**Moderate**				**Severe**				**Extremely severe**			
	**OR**	***P* value**	**95%** ***CI***		**OR**	***P* value**	**95%** ***CI***		**OR**	***P* value**	**95%** ***CI***		**OR**	***P* value**	**95%** ***CI***	
			**Lower**	**Upper**			**Lower**	**Upper**			**Lower**	**Upper**			**Lower**	**Upper**
**Mental effect of pandemic (Ref: No)**																
Yes	**1.74**	0.00	1.18	2.54	**2.25**	0.00	1.60	3.16	**3.38**	0.00	2.28	5.49	**3.70**	0.00	2.48	5.52
Maybe	1.33	0.16	0.89	1.99	**1.51**	0.02	1.05	2.18	1.42	1.77	0.85	2.37	**1.55**	0.05	0.98	2.45
**New cases and deaths (Ref: None)**																
Fear	**2.48**	0.03	1.04	5.90	**4.25**	0.00	1.93	9.37	**2.34**	0.04	1.01	5.39	**2.19**	0.05	0.99	4.81
Depressed	2.12	0.05	0.97	4.64	**2.63**	0.01	1.25	5.52	2.03	0.07	0.94	4.38	**2.20**	0.03	1.07	4.51
Worried	**2.69**	0.00	1.34	5.37	**3.15**	0.00	1.59	6.21	1.41	0.34	0.68	2.90	0.97	0.93	0.48	1.94
Anxious	**2.71**	0.01	1.21	6.05	**3.39**	0.00	1.58	7.30	1.08	0.86	0.44	2.60	1.77	0.14	0.82	3.83
Upset	1.72	0.12	0.85	3.47	**1.99**	0.05	1.00	3.98	0.83	0.63	0.39	1.76	0.88	0.74	0.44	1.79
**Time on social media (Ref: < 1 h)**																
1–3h	1.34	0.14	0.90	1.97	1.39	0.06	0.99	1.95	1.58	0.07	1.07	2.34	1.30	0.15	0.90	1.87
4–6h	0.73	0.51	0.29	1.85	1.45	0.24	0.76	2.77	1.04	0.91	0.46	2.38	1.33	0.40	0.67	2.66
More than 6 h	1.52	0.48	0.46	4.94	1.70	0.31	0.60	4.80	**4.56**	0.00	1.74	11.95	**9.01**	0.00	3.86	21.03
**Effect of lockdown (Ref: None)**																
Fear	1.38	0.56	0.46	4.11	1.82	0.18	0.75	4.43	**2.59**	0.05	0.97	6.91	**7.98**	0.00	3.12	20.43
Depressed	**2.37**	0.00	1.43	3.91	**3.74**	0.00	2.40	5.82	**4.37**	0.00	2.49	7.65	**17.77**	0.00	9.58	32.95
Worried	1.54	0.07	0.95	2.49	**2.43**	0.00	1.60	3.70	**2.12**	0.00	1.20	3.73	**4.89**	0.000	2.59	9.24
Anxious	**2.23**	0.00	1.41	3.52	**2.48**	0.00	1.63	3.78	**2.61**	0.00	1.50	4.55	**6.80**	0.00	3.66	12.65
Upset	**2.04**	0.00	1.23	3.39	**2.03**	0.00	1.26	3.27	**2.68**	0.00	1.46	4.89	**5.50**	0.00	2.82	10.72
**Survival chances (Ref: I will get well soon)**																
Will not survive	3.23	0.11	0.73	14.19	2.07	0.34	0.45	9.37	**5.05**	0.02	1.27	19.99	**8.17**	0.00	2.24	29.77
Not sure	0.89	0.55	0.62	1.28	1.29	0.08	0.96	1.73	1.21	0.27	0.85	1.73	**1.76**	0.00	1.29	2.39

*p < 0.05 is represented with **bold***.

As per Table 4B (Anxiety), the factor “mental effect of pandemic” increases the likelihood of anxiety for the respondents who believe that the pandemic has affected them mentally and those who remains neutral regarding the mental effect of pandemic with mild (OR affected = 2.67, OR neutral = 1.98), moderate (OR affected = 2.11, OR neutral = 1.68), severe (OR affected = 4.00), and extremely severe states (OR affected = 4.78) of anxiety. In contrast to those who does not report any effect of “new COVID-19 cases and deaths,” an increased likelihood of having anxiety is observed for the respondents who fear, depressed, worried, and anxious with moderate (OR fear = 3.60, OR depressed = 3.06, OR worried= 2.41, OR anxious = 2.32) and extremely severe (OR fear = 2.58, OR depressed = 3.52) states of anxiety. It is also observed that the likelihood of anxiety increases for the respondents who fear, depressed, worried, anxious, and upset from the “implication of lockdown” evident with mild (OR fear = 3.34), moderate (OR fear = 5.33, OR depressed = 2.82, OR worried = 1.71, OR anxious = 2.40, OR upset = 1.78), severe (OR fear = 4.75, OR anxious = 2.28), and extremely severe (OR fear = 9.24, OR depressed = 5.06, OR worried = 2.13, OR anxious = 3.57, OR upset = 2.55) states of anxiety, as opposed to those with no affect. The increase in “time spent on social media” to gather COVID-19 updates increases the likelihood of extremely severe (OR = 5.12) state of anxiety. When compared with those who believe that they will “survive the COVID-19 infection,” the respondents who believe that they will have less or no survival probability are at higher likelihood of moderate (OR not survive = 3.74, OR not sure = 1.31), severe (OR not survive = 6.71, OR not sure = 1.63), and extremely severe (OR not survive = 8.86, OR not sure = 1.57) state anxiety.

**Table 4B:** Association of level of severity of anxiety with the personal feeling variable identified with multinomial logistic regression.

**Anxiety**																
	**Mild**				**Moderate**				**Severe**				**Extremely Severe**			
	**OR**	***P*** **value**	**95%** ***CI***		**OR**	***P*** **value**	**95%** ***CI***		**OR**	***P*** **value**	**95%** ***CI***		**OR**	**P value**	**95%** ***CI***	
			**Lower**	**Upper**			**Lower**	**Upper**			**Lower**	**Upper**			**Lower**	**Upper**
**Mental effect of pandemic (Ref: No)**																
Yes	**2.67**	0.00	1.72	4.16	**2.11**	0.00	1.49	2.97	**4.00**	0.00	2.23	7.174	**4.78**	0.00	3.08	7.43
Maybe	**1.98**	0.00	1.25	3.15	**1.68**	0.00	1.16	2.43	1.10	0.77	0.53	2.286	1.47	0.15	0.86	2.49
**New cases and deaths (Ref: None)**																
Fear	1.97	0.11	0.84	4.62	**3.60**	0.00	1.62	7.95	2.52	0.07	0.912	6.98	**2.58**	0.03	1.07	6.23
Depressed	1.38	0.42	0.62	3.05	**3.06**	0.00	1.45	6.44	1.45	0.45	0.540	3.94	**3.52**	0.00	1.54	8.01
Worried	1.20	0.61	0.59	2.44	**2.41**	0.01	1.19	4.87	1.06	0.89	0.419	2.71	1.03	0.93	0.45	2.33
Anxious	1.05	0.90	0.43	2.55	**2.32**	0.03	1.05	5.09	1.37	0.55	0.482	3.89	2.05	0.10	0.85	4.90
Upset	1.68	0.14	0.83	3.40	1.95	0.06	0.95	3.99	0.82	0.69	0.311	2.18	1.28	0.54	0.56	2.93
**Time on social media (Ref: < 1 h)**																
1–3 h	0.91	0.69	0.58	1.43	1.25	0.15	0.91	1.72	1.46	0.09	0.931	2.29	1.13	0.47	0.79	1.62
4–6 h	2.02	0.05	0.97	4.21	1.33	0.40	0.68	2.61	1.74	0.22	0.719	4.21	1.42	0.32	0.70	2.85
More than 6 h	2.48	0.06	0.94	6.50	2.02	0.10	0.87	4.72	2.03	0.28	0.558	7.38	**5.12**	0.00	2.33	11.21
**Effect of lockdown (Ref: None)**																
Fear	**3.34**	0.01	1.23	9.05	**5.33**	0.00	2.22	12.80	**4.75**	0.00	1.504	15.00	**9.24**	0.00	3.48	24.55
Depressed	1.21	0.45	0.73	2.02	**2.82**	0.00	1.81	4.39	1.84	0.09	0.900	3.76	**5.06**	0.00	2.81	9.10
Worried	0.81	0.44	0.48	1.37	**1.71**	0.01	1.09	2.68	1.78	0.10	0.881	3.60	**2.13**	0.01	1.14	3.97
Anxious	0.92	0.76	0.55	1.54	**2.40**	0.00	1.55	3.72	**2.28**	0.01	1.146	4.54	**3.57**	0.00	1.97	6.47
Upset	0.91	0.74	0.52	1.58	**1.78**	0.02	1.09	2.90	1.03	0.93	0.440	2.43	**2.55**	0.00	1.33	4.88
**Survival chances (Ref: I will get well soon)**																
Will not survive	0.88	0.91	0.10	7.64	**3.74**	0.02	1.15	12.13	**6.71**	0.00	1.824	24.72	**8.86**	0.00	3.00	26.12
Not sure	0.98	0.91	0.67	1.43	**1.31**	0.04	0.99	1.74	**1.63**	0.01	1.086	2.45	**1.57**	0.00	1.16	2.14

*p < 0.05 is represented with **bold***.

As per Table 4C (Stress), the factor “mental effect of pandemic” increases the likelihood of stress for the respondents who believe that the pandemic has affected them mentally with mild (OR affected = 1.59), moderate (OR affected = 2.52), severe (OR affected = 4.35), and extremely severe states (OR affected = 6.07) of stress. In contrast to those who does not report any effect of “new COVID-19 cases and deaths,” an increased likelihood of having stress is observed for the respondents who fear and depressed, with mild (OR fear = 2.32) and moderate (OR fear = 2.85, OR depressed = 2.33) states of stress. It is also observed that the likelihood of stress increases for the respondents who fear, depressed, worried, anxious, and upset from the “implication of lockdown” evident with mild (OR depressed = 3.06, OR anxious = 1.80), moderate (OR fear = 2.56, OR depressed = 3.52, OR worried = 1.68, OR anxious = 2.95, OR upset = 1.87), severe (OR fear = 6.99, OR depressed = 6.30, OR worried = 2.76, OR anxious = 4.39, OR upset = 2.65), and extremely severe (OR fear = 10.31, OR depressed = 11.33, OR worried = 3.75, OR anxious = 6.48) state of stress, as opposed to those with no affect. The increase in “time spent on social media” to gather COVID-19 updates increases the likelihood of mild (OR = 2.25), moderate (OR = 3.57), severe (OR = 3.28), and extremely severe (OR = 4.39) state of stress. When compared with those who believe that they will “survive the COVID-19 infection,” the respondents who believe that they will have less or no survival probability are at higher likelihood of mild (OR not survive = 4.42, OR not sure = 1.40), moderate (OR not sure = 1.47), severe (OR not sure = 1.56), and extremely severe (OR not survive = 15.66, OR not sure = 2.02) state stress.

**Table 4C:** Association of level of severity of stress with the personal feeling variable identified with multinomial logistic regression.

**Stress**																
	**Mild**				**Moderate**				**Severe**				**Extremely severe**			
	**OR**	***P*** **value**	**95%** ***CI***		**OR**	***P*** **value**	**95%** ***CI***		**OR**	***P*** **value**	**95%** ***CI***		**OR**	***P*** **value**	**95%** ***CI***	
			**Lower**	**Upper**			**Lower**	**Upper**			**Lower**	**Upper**			**Lower**	**Upper**
**Mental effect of pandemic (Ref: No)**																
Yes	**1.59**	0.00	1.17	2.16	**2.52**	0.00	1.70	3.71	**4.35**	0.00	2.64	7.15	**6.07**	0.00	3.17	11.64
Maybe	1.23	0.21	0.88	1.70	1.43	0.10	0.92	2.20	1.20	0.55	0.65	2.21	1.82	0.12	0.85	3.89
**New cases and deaths (Ref: None)**																
Fear	**2.32**	0.00	1.25	4.29	**2.85**	0.01	1.25	6.49	1.37	0.52	0.51	3.69	2.46	0.13	0.75	8.01
Depressed	0.98	0.95	0.54	1.75	**2.33**	0.03	1.08	5.01	2.37	0.05	0.98	5.76	2.82	0.06	0.92	8.62
Worried	1.37	0.20	0.83	2.27	1.95	0.06	0.94	4.01	0.94	0.89	0.39	2.25	0.77	0.66	0.25	2.39
Anxious	1.67	0.08	0.92	3.04	2.11	0.07	0.93	4.79	1.54	0.37	0.59	4.04	2.00	0.24	0.61	6.54
Upset	1.11	0.68	0.66	1.84	1.02	0.95	0.48	2.17	1.02	0.95	0.42	2.48	1.05	0.92	0.34	3.27
**Time on social media (Ref: < 1 h)**																
1–3 h	0.97	0.88	0.71	1.34	1.30	0.13	0.92	1.84	1.19	0.38	0.79	1.78	0.78	0.30	0.48	1.25
4–6 h	1.10	0.75	0.58	2.10	1.34	0.41	0.66	2.70	1.50	0.28	0.71	3.16	0.88	0.80	0.35	2.25
More than 6 h	**2.25**	0.04	1.02	4.96	**3.57**	0.00	1.53	8.30	**3.28**	0.01	1.22	8.82	**4.39**	0.00	1.62	11.90
**Effect of lockdown (Ref: None)**																
Fear	1.59	0.28	0.67	3.77	**2.56**	0.04	1.00	6.54	**6.99**	0.00	2.48	19.67	**10.31**	0.00	3.04	34.94
Depressed	**3.06**	0.00	2.07	4.50	**3.52**	0.00	2.13	5.81	**6.30**	0.00	3.16	12.53	**11.33**	0.00	4.58	28.01
Worried	1.40	0.08	0.95	2.06	**1.68**	0.04	1.01	2.79	**2.76**	0.00	1.35	5.63	**3.75**	0.00	1.46	9.65
Anxious	**1.80**	0.00	1.23	2.63	**2.95**	0.00	1.81	4.79	**4.39**	0.00	2.21	8.74	**6.48**	0.00	2.59	16.20
Upset	1.50	0.05	0.99	2.28	**1.87**	0.02	1.08	3.26	**2.65**	0.01	1.23	5.68	2.58	0.07	0.92	7.23
**Survival chances (Ref: I will get well soon)**																
Will not survive	**4.42**	0.01	1.42	13.76	2.57	0.18	0.63	10.39	1.65	0.56	0.29	9.26	**15.66**	0.00	4.80	51.11
Not sure	**1.40**	0.01	1.07	1.84	**1.47**	0.01	1.08	2.01	**1.56**	0.01	1.09	2.22	**2.02**	0.00	1.37	2.97

*p < 0.05 is represented with **bold***.

## Discussion

The aim of this study is to investigate the mental health status of general public of Pakistan and the level of severity of emotional states of depression, anxiety, and stress in the aftermath of COVID-19 based on demographic, socioeconomic, and psychological variables. This study found that 60.9, 48.1, and 53.4% of the respondents were symptomatic with 29.8, 21.7, and 18.3% severe and extremely severe cases for depression, anxiety, and stress. This is consistent with a previous study indicating 35% prevalence rate of depression in CI Karachi, Pakistan. Moreover, another study in Karachi, Pakistan, along with various other provincial areas indicates <2% of extreme emotional state as measured by DASS before COVID-19 pandemic (36–41). These increased levels of psychological distress evident from this study could therefore be perceived as the impact of COVID-19 pandemic.

The findings from our demographic data revealed gender, age, and marital status to be a strong predictor of DASS subscales. A greater psychological distress was observed for females and unmarried population under the age of 30 and is coherent with the previous studies (1, 59–63). Increased depression, anxiety, and stress levels in unmarried population could be explained with the fact that Pakistan is a country with a dominant population of young adults, most of which are unmarried or single for up to 30 years of age (35, 64, 65). These findings recommend the government to take serious attention to conduct awareness sessions and to take initiatives for the assessment of the mental health of young individuals under the age of 30 which are identified to be more prone to increasing level of severity of depression, anxiety, and stress and provide psychological interventions to the identified high-risk group, thereby targeting majority of Pakistani population.

The government of Pakistan took initiative to support small-scale business and for providing financial aid in order to ensure food supplies for those having large household size and for those whose earnings are reduced during this pandemic. These programs include Ehsaas Koye Bhooka Na Soye (EKBNS) and Kamyab Pakistan Program (KPP) (66, 67). This study reported that earnings and household size are strong predictor of depression, which is in accordance with other studies conducted in pandemic (62, 68, 69). Hence, it is recommended that government should not only continue but expand those programs because controlling mental health issues due to socioeconomic predictors requires financial assistance from state. These initiatives would have an impact even after pandemic.

The most notable finding of this study is that the outbreak of COVID-19 pandemic induces fear in general public of Pakistan primarily in the target population that spend more time on social media to obtain updated information related to rise in COVID-19 infected cases, misconception of lower survival possibility upon acquiring COVID-19 infection and escalated mortality rate and complies with the existing literature (70–72). This adverse psychological impact due to electronic and social media could be explained with 77% population using smartphones and 63% of the 76 million people who have Internet access (18, 73, 74). It is further supported from the existing literature which indicated that the excessive Internet use was significantly associated with depression and anxiety (75) and social media use was a significant factor in higher problematic smartphone use (76). The academic institutes in particular universities majorly comprise of young population together with the support of research centers will allow the clinicians to offer digitize assessment and up-to-date technology-based treatment to the majority of the most vulnerable group of Pakistani population. The engineers and researchers at research centers can develop and implement the state-of-the-art technology that could transform the traditional clinical practices and enable the psychologist and psychotherapist to conduct multiple treatment sessions simultaneously thereby reducing their load. To support the limited number of psychologist and psychiatrist (i.e., 0.48 per 100,000 population), the neurocomputation research group is working in close collaboration with clinical psychologist to deliver effective technological solutions such as transcranial direct current stimulation, neurofeedback, and binaural beat necessary to reduce the psychological sufferings of the general population of Pakistan without burdening the healthcare practitioners. A total of 40 respondents were provided with the technology-based treatment by the neurocomputation research group at the request of clinical practitioners, out of 120 respondents who visited the mental healthcare professionals *via* this survey. The preliminary findings of technology-based treatment are promising, and clinicians showed comfort for practicing in clinic. The availability of technology-based treatment will reduce the treatment load of mental healthcare providers so that they could concentrate more on assessment of the mental wellbeing of the respondents thereby managing the assessment load. As indicated in this study, where COVID-19 induced depression, anxiety, and stress is at its peak, it is highly needed that similar model with interdisciplinary field should work collectively and also ensure its practical implementation for better management of mental health issues in a country where mental health is considered a social stigma and have limited number of psychologist and psychotherapist to provide medical attention to the affected individuals. Hence, this study will serve as a model for other clinics and research centers to revolutionize the conventional clinical practices.

This study has several limitations. It includes online responses since population-based survey was not possible during the COVID-19 outbreak. The online response has limited the accessibility of the survey to the respondents having an Internet facility which is largely available in the urban centers of Pakistan. The non-urban areas of Pakistan have limited Internet accessibility because of which a relatively small number of responses were gathered. This reduces the generalizability and increases the selection bias, of our model. However, the usability of Internet has enabled collection of large data. Moreover, the population of Karachi have diversified origins and cover people from both rural and urban regions (77–79) thereby ensuring generalizability of the study to the general population of Pakistan and in particular to the urban center of Pakistan. Most of the respondents were educated, thus skewing results of an entire population to solely college-educated civilians and not those with other forms of education or work. Oversampling of young participants may also cause bias and limits its applicability to the entire population. However, the distribution of Pakistan population is skewed toward young age under 30 as indicated by the Pakistan Bureau of Statistics (35). As this is an online research study, the responses obtained from the respondents under 18 years of age could be argued. Moreover, with online survey, a Hawthorne effect, that is the feeling that someone is analyzing these results, may skew the results to be more favorable one way or another. Future research is needed to include people from rural areas, those with a less than college education, as well as to further increase the generalizability of the data collected.

## Conclusion

This study suggests that variable of personal feeling has significantly impacted the mental wellbeing and has largely induced increasing severity levels of COVID-19 related depression, anxiety, and stress. Hence, it is required to conduct awareness seminars, assessment, and treatment sessions on large scale. This indicates the need of collaborative initiatives of technology-based treatment so that the limited number of mental health practitioners could cope up with the increasing number of patients with COVID-19 induced depression, anxiety, and stress.

## Data Availability Statement

The raw data supporting the conclusions of this article will be made available by the authors, without undue reservation.

## Ethics Statement

The studies involving human participants were reviewed and approved by Nadirshaw Eduljee Dinshaw (NED) University of Engineering and Technology's Ethical Review Committee in Karachi, Pakistan. Written informed consent to participate in this study was provided by the participants' legal guardian/next of kin.

## Author Contributions

MH, HK, SQ, MI, SA, and MA conceptualized the study. MH, HS, and OE designed methodology. HS, OE, and MI are involved in formal analysis. MH, MI, MA, SA, HS, OE, and HK investigated the study. MH, HK, and SQ collected resources. MH and SQ are involved in data curation and funding acquisition and supervised the study. HS was involved in writing and original draft preparation. HS and SA wrote the manuscript. OE and HS visualized the study. SQ took a leading role in project administration. All authors have read and agreed to the published version of the manuscript.

## Funding

This research was funded by the World Bank, Grant Number 7197087.

## Conflict of Interest

MI and MA were employed by Al'Shakoor Foundation. The remaining authors declare that the research was conducted in the absence of any commercial or financial relationships that could be construed as a potential conflict of interest.

## Publisher's Note

All claims expressed in this article are solely those of the authors and do not necessarily represent those of their affiliated organizations, or those of the publisher, the editors and the reviewers. Any product that may be evaluated in this article, or claim that may be made by its manufacturer, is not guaranteed or endorsed by the publisher.

## References

[1] Wang C Pan R Wan X Tan Y Xu L Ho CS. Immediate psychological responses and associated factors during the initial stage of the 2019 coronavirus disease (COVID-19) epidemic among the general population in China. Int J Env Res Public Health. (2019) 17:1729. 10.3390/ijerph17051729 32155789 PMC7084952

[2] Zhu N Zhang D Wang W Li X Yang B Song J. A novel coronavirus from patients with pneumonia in China, 2019. N Engl J Med. (2020) 382:727–33. 10.1056/NEJMoa2001017 31978945 PMC7092803

[3] Communication S Ahmed N Maqsood A Abduljabbar T Vohra F. Tobacco smoking a potential risk factor in transmission of COVID-19 infection. Pakistan J Med Sci. (2020) 36:104–7. 10.12669/pjms.36.COVID19-S4.2739 32582324 PMC7306971

[4] Anita P Jernigan DB. Initial Public Health Response and Interim Clinical Guidance for the 2019 Novel Coronavirus Outbreak—United States, 31 December 2019–4 February 2020. Morb Mortal Wkly Rep. (2020) 69:140–6. 10.15585/mmwr.mm6905e1 32027631 PMC7004396

[5] Nishiura H Jung S Linton NM Kinoshita R Yang Y Hayashi K. The extent of transmission of novel coronavirus inWuhan, China, 2020. J Clin Med. (2020). 10.3390/jcm9020330 31991628 PMC7073674

[6] Azim D Nasim S Kumar S Hussain A Jaipal M. Media on the frontline against mental health implications of COVID-19 in Pakistan. Asian J Psychiatr. (2020) 54:102342. 10.1016/j.ajp.2020.102342 32763525 PMC7387925

[7] Ilyas N Azuine RE Tamiz A. COVID-19 Pandemic in Pakistan. Int J Transl Med Res Public Health. (2020) 4:37–49. 10.21106/ijtmrph.139

[8] Xiao H Zhang Y Kong D Li S Yang N. The effects of social support on sleep quality of medical staff treating patients with coronavirus disease 2019(COVID-19) in January and February 2020 in China. Med Sci Monit. (2020) 26:1–8. 10.12659/MSM.923549 32132521 PMC7075079

[9] Rajkumar RP. COVID-19 and mental health: a review of the existing literature. Asian J Psychiatry J. (2020) 52:102066. 10.1016/j.ajp.2020.102066 32302935 PMC7151415

[10] Hall RCW D M Hall RCW D M Chapman MJ. The 1995 Kikwit Ebola outbreak: lessons hospitals and physicians can apply to future viral epidemics. Gen Hosp Psychiatry. (2008) 30:446–52. 10.1016/j.genhosppsych.2008.05.003 18774428 PMC7132410

[11] Sim K Huak Y Nah P Choon H Wen S. Psychosocial and coping responses within the community health care setting towards a national outbreak of an infectious disease. J Psychosom Res. (2010) 68:195–202. 10.1016/j.jpsychores.2009.04.004 20105703 PMC7094450

[12] Tandon R. The COVID-19 pandemic, personal reflections on editorial responsibility. Asian J Psychiatr. (2020) 50:102100. 10.1016/j.ajp.2020.102100 32354694 PMC7165287

[13] Paul KI Moser K. Unemployment impairs mental health: meta-analyses. J Vocat Behav. (2009) 74:264–82. 10.1016/j.jvb.2009.01.001

[14] Mamun MA Ullah I. COVID-19 suicides in Pakistan, dying off not COVID-19 fear but poverty? The forthcoming economic challenges for a developing country. Brain Behav Immun. (2020) 2020:1–4. 10.1016/j.bbi.2020.05.028 32407859 PMC7212955

[15] Oyesanya M Lopez-Morinigo J Dutta R. Systematic review of suicide in economic recession. World J Psychiatry. (2015) 5:243. 10.5498/wjp.v5.i2.243 26110126 PMC4473496

[16] Kawohl W Nordt C. COVID-19, unemployment, and suicide. Lancet Psychiatry. (2020) 7:389–90. 10.1016/S2215-0366(20)30141-3 32353269 PMC7185950

[17] Haider II Tiwana F Tahir SM. Impact of the COVID-19 pandemic on adult mental health. Pak J Med Sci. (2020) 36:S90–4. This. 10.12669/pjms.36.COVID19-S4.2756 32582321 PMC7306943

[18] Saqlain M Ahmed A Gulzar A Naz S Munir MM Kamran S. Public's knowledge and practices regarding COVID-19: a cross-sectional survey from Pakistan. MedRxiv. (2020). 10.1101/2020.06.01.20119404 34026708 PMC8133216

[19] Zhao N Zhou G. Social media use and mental health during the COVID-19 pandemic: moderator role of disaster stressor and mediator role of negative affect. Appl Psychol Health Well Being. (2020) 12:1019–38. 10.1111/aphw.12226 32945123 PMC7536964

[20] Zhang YT Li RT Sun XJ Peng M Li X. Social media exposure, psychological distress, emotion regulation, and depression during the COVID-19 outbreak in community samples in China. Front Psychiatry. (2021) 12:644899. 10.3389/fpsyt.2021.644899 34054602 PMC8149733

[21] Hosen I Al-Mamun F Mamun MA. Prevalence and risk factors of the symptoms of depression, anxiety, and stress during the COVID-19 pandemic in Bangladesh: a systematic review and meta-analysis. Glob Ment Health. (2021) 8:e47. 10.1017/gmh.2021.49 35145709 PMC8794743

[22] Mamun MA Misti JM Hosen I Al Mamun F. Suicidal behaviors and university entrance test-related factors: a Bangladeshi exploratory study. Perspect Psychiatr Care. (2022) 58:278–87. 10.1111/ppc.12783 33834493

[23] Mamun MA Safiq MB Hosen I Mamun F. Suicidal behavior and flood effects in Bangladesh: a two-site interview study. Risk Manag Healthc Policy. (2021) 14:129–44. 10.2147/RMHP.S282965 33469396 PMC7812054

[24] Al Mamun F Gozal D Hosen I Misti JM Mamun MA. Predictive factors of insomnia during the COVID-19 pandemic in Bangladesh: a GIS-based nationwide distribution. Sleep Med. (2022) 91:219–25. 10.1016/j.sleep.2021.04.025 33975776 PMC9017957

[25] Al Mamun F Hosen I Misti JM Kaggwa MM Mamun MA. Mental disorders of Bangladeshi students during the COVID-19 pandemic: a systematic review. Psychol Res Behav Manag. (2021) 14:645–54. 10.2147/PRBM.S315961 34104010 PMC8180282

[26] Casagrande M Favieri F Tambelli R Forte G. The enemy who sealed the world: effects quarantine due to the COVID-19 on sleep quality, anxiety, and psychological distress in the Italian population. Sleep Med. (2020) 75:12–20. 10.1016/j.sleep.2020.05.011 32853913 PMC7215153

[27] Forte G Favieri F Tambelli R Casagrande M. The enemy which sealed the world: effects of COVID-19 diffusion on the psychological state of the Italian population. J Clin Med. (2020) 9:1802. 10.3390/jcm9061802 32531884 PMC7356935

[28] Forte G Favieri F Tambelli R Casagrande M. COVID-19 pandemic in the Italian population: validation of a post-traumatic stress disorder questionnaire and prevalence of PTSD symptomatology. Int J Environ Res Public Health. (2020) 17:1–16. 10.3390/ijerph17114151 32532077 PMC7312976

[29] Choi EPH Hui BPH Wan EYF. Depression and anxiety in Hong Kong during covid-19. Int J Environ Res Public Health. (2020) 17:3740. 10.3390/ijerph17103740 32466251 PMC7277420

[30] Hyland P Shevlin M McBride O Murphy J Karatzias T Bentall RP. Anxiety and depression in the Republic of Ireland during the COVID-19 pandemic. Acta Psychiatr Scand. (2020) 142:249–56. 10.1111/acps.13219 32716520

[31] Özdin S Bayrak Özdin S. Levels and predictors of anxiety, depression and health anxiety during COVID-19 pandemic in Turkish society: the importance of gender. Int J Soc Psychiatry. (2020) 66:504–11. 10.1177/0020764020927051 32380879 PMC7405629

[32] Gallagher MW Zvolensky MJ Long LJ Rogers AH Garey L. The impact of Covid-19 experiences and associated stress on anxiety, depression, and functional impairment in American adults. Cogn Ther Res. (2020) 44:1043–51. 10.1007/s10608-020-10143-y 32904454 PMC7456202

[33] Rubin GJ Potts HWW Michie S. The impact of communications about swine flu (influenza A H1N1v) on public responses to the outbreak : results from 36 national telephone surveys in the UK. Health Technol Assess. (2010) 14:183–266. 10.3310/hta14340-03 20630124

[34] World Health Organization and Ministry of Health. WHO-AIMS Report on Mental Health System in Pakistan. World Health Organization (2009). Available online at: http://www.who.int/mental_health/pakistan_who_aims_report.pdf

[35] Pakistan Bureau of Statistics. Population By 5 Year Age Groups—Pakistan. Gov Pakistan (n.d). Available online at: https://www.pbs.gov.pk (accessed September 15, 2020).

[36] Syed A Ali SS Khan M. Frequency of depression, anxiety and stress among the undergraduate physiotherapy students. Pakistan J Med Sci. (2018) 34:468–71. 10.12669/pjms.342.12298 29805428 PMC5954399

[37] Azim SR Baig M. Frequency and perceived causes of depression, anxiety and stress among medical students of a private medical institute in karachi: a mixed method study. J Pak Med Assoc. (2019) 69:840–5. 10.4135/9781529734348 31189292

[38] Rizvi F Qureshi A Rajput A Afzal M. Prevalence of depression, anxiety and stress (by DASS scoring system) among medical students in Islamabad, Pakistan. Br J Med Med Res. (2015) 8:69–75. 10.9734/BJMMR/2015/17193

[39] Kumar H. Psychological distress and life satisfaction among university students. J Psychol Clin Psychiatry. (2016) 5:1–8. 10.15406/jpcpy.2016.05.00283

[40] Naqshbandi I Bashir N Qadri SY Khan SS. Prevalence of stress, anxiety and depression among medical undergraduate students of Kashmir—a cross-sectional study. Int J Contemp Med Res. (2019) 6:6–8. 10.21276/ijcmr.2019.6.5.40

[41] Sehrish H Waqar H. The different levels of depression and anxiety among Pakistani professionals. Insights Depress Anxiety. (2020) 4:012–8. 10.29328/journal.ida.1001014

[42] Districtwise Statistics COVID 19. Health Department of Sindh (2022). Available online at:https://sindhhealth.gov.pk/Districtwise-Statistics-COVID-19 (accessed November 11, 2021).

[43] Field MJ Behrman RE, Children Children of M (US) C on CRI. Understanding and Agreeing to Children's Participation in Clinical Research. Institute of Medicine (US) Committee on Clinical Research Involving Children (2004).

[44] *List of Largest Cities in Pakistan* (2022). Available online at: https://zims-en.kiwix.campusafrica.gos.orange.com/Wikipedia-en-all-nopic/A/List-of-largest-cities-in-Pakistan (accessed February 7, 2022).

[45] About Karachi (2022). Available online at: http://www.kmc.gos.pk/contents.aspx?id=14 (accessed February 7, 2022).

[46] Details I. COVID-19 Situation ! Pakistan Statistics Province Wise Cases. Ministry of National Health Services Regulations & Coordination (2021). p. 1–7. Available online at: http://covid.gov.pk/

[47] Resnik DB Jones CW, Research subjects with limited english proficiency. Ethical and legal issues. Account Res. (2006) 13:157–77. 10.1080/08989620600654043 16830406 PMC3942994

[48] Sha M Immerwahr S. Survey translation: why and how should researchers and managers be engaged? Surv Pract. (2018) 11:1–10. 10.29115/SP-2018-0016

[49] Junaid K Daood M Saleem S Nazim R Rs S. Prevalence and associated factors of depression, anxiety, and stress among the general population during COVID-19 pandemic: cross-sectional study in Lahore, Pakistan. Med J Clin Trials Case Stud. (2021) 5:000S1–013. 10.23880/mjccs-16000S1-01333627000

[50] Suneel DI Schwaiger DE Nazim DA Khaqan S Khan AY Munir M. Psychological health and its correlates during the COVID-19 pandemic in Pakistan: a survey of undergraduate students. J Prof Appl Psychol. (2021) 2:156–69. 10.52053/jpap.v2i2.661

[51] Arshad MS Hussain I Nafees M Majeed A Imran I Saeed H. Assessing the impact of COVID-19 on the mental health of healthcare workers in three metropolitan cities of Pakistan. Psychol Res Behav Manag. (2020) 13:1047–55. 10.2147/PRBM.S282069 33244279 PMC7685388

[52] Hayat K Haq MIU Wang W Khan FU Rehman A Rasool MF. Impact of the COVID-19 outbreak on mental health status and associated factors among general population: a cross-sectional study from Pakistan. Psychol Health Med. (2022) 27:54–68. 10.1080/13548506.2021.1884274 33627000

[53] Galea S Ettman CK. Mental health and mortality in a time of COVID-19. Am J Public Health. (2021) 111:S73–4. 10.2105/AJPH.2021.306278 34314215 PMC8495645

[54] Mumtaz M. COVID-19 and mental health challenges in Pakistan. Int J Soc Psychiatry. (2021) 67:303–4. 10.1177/0020764020954487 32900252

[55] Zhang T. Correlations between COVID-19 case growth and mental health-related internet search: an unexpected finding. Iran J Public Health. (2020) 49:2001–2. 10.18502/ijph.v49i10.4706 33346206 PMC7719640

[56] Mazza C Ricci E Biondi S Colasanti M Ferracuti S Napoli C. A nationwide survey of psychological distress among Italian people during the COVID-19 pandemic: immediate psychological responses and associated factors. Int J Environ Res Public Health. (2020) 17:3165. 10.3390/ijerph17093165 32370116 PMC7246819

[57] Lee AM Wong JGWS McAlonan GM Cheung V Cheung C Sham PC. Stress and psychological distress among SARS survivors 1 year after the outbreak. Can J Psychiatry. (2007) 52:233–40. 10.1177/070674370705200405 17500304

[58] McAlonan GM Lee AM Cheung V Cheung C Tsang KWT Sham PC. Immediate and sustained psychological impact of an emerging infectious disease outbreak on health care workers. Can J Psychiatry. (2007) 52:241–7. 10.1177/070674370705200406 17500305

[59] Qiu J Shen B Zhao M Wang Z Xie B Xu Y. Nationwide survey of psychological distress among Chinese people in the COVID-19 epidemic : implications and policy recommendations. Gen Psychiatry. (2020) 33:e100213. 10.1136/gpsych-2020-100213 32215365 PMC7061893

[60] Ryba MM Hopko DR. Gender differences in depression: assessing mediational effects of overt behaviors and environmental reward through daily diary monitoring. Depress Res Treat. (2012) 2012:865679. 10.1155/2012/865679 22454769 PMC3290799

[61] Kidwai R. Demographic factors, social problems and material amenities as predictors of psychological distress: a cross-sectional study in Karachi, Pakistan. Soc Psychiatry Psychiatr Epidemiol. (2014) 49:27–39. 10.1007/s00127-013-0692-0 23620098

[62] Sareen J Afifi TO McMillan KA Asmundson GJG. Relationship between household income and mental disorders. Arch Gen Psychiatry. (2011) 68:419–27. 10.1001/archgenpsychiatry.2011.15 21464366

[63] Husain N Chaudhry N Jafri F Tomenson B Surhand I Mirza I. Prevalence and risk factors for psychological distress and functional disability in urban Pakistan. WHO South-East Asia J Public Health. (2014) 3:144–53. 10.4103/2224-3151.206730 28607300

[64] Pakistan Pakistan Bureau of Statistics, Gov Pakistan. Population (15 Years and Above) By Marital Status (2018). Available online at: https://www.pbs.gov.pk (accessed September 15, 2020).

[65] Pakistan Bureau of Statistics, Gov Pakistan. Population by Selective Age Groups (2018). Available online at:www.pbs.gov.pk (accessed September 15, 2020).

[66] Ehsaas (2022). Available online at: https://www.pass.gov.pk/Detail9a774dca-e5d6-4ad5-9a08-f425a686559d (accessed December 14, 2021).

[67] Kamyab Jawan (2022). Available online at: https://kamyabjawan.gov.pk/ (accessed December 14, 2021).

[68] Lateef T Chen J Tahir M Lateef TA Chen BZ Li J. Typhoon eye effect versus ripple effect: the role of family size on mental health during the COVID-19 pandemic in Pakistan. Glob Heal. (2021) 17:32. 10.1186/s12992-021-00685-5 33781286 PMC8006139

[69] M K G W. Economics and mental health: the current scenario. World Psychiatry. (2020) 19:3–14. 10.1002/wps.20692 31922693 PMC6953559

[70] Wu KK Chan SK Ma TM. Posttraumatic stress, anxiety, and depression in survivors of severe acute respiratory syndrome (SARS). J Trauma Stress. (2005) 18:39–42. 10.1002/jts.20004 16281194 PMC7166878

[71] Xiao C A. Novel approach of consultation on 2019 novel coronavirus (COVID-19) -related psychological and mental problems : structured letter therapy. Psychiatry Investig. (2020) 17:175–6. 10.30773/pi.2020.0047 32093461 PMC7047000

[72] Mak VWC Chu CM Pan PC Yiu MGC Chan. VL. Long-term psychiatric morbidities among SARS survivors *Gen Hosp Psychiatry*. (2009) 31:318–26. 10.1016/j.genhosppsych.2009.03.001 19555791 PMC7112501

[73] Pakistan Telecommunication Authority. Annual Report: 2014. Islamabad: Pakistan Telecommunication Authority (2014). Available online at: https://www.pta.gov.pk/annual-reports/ptaannrep2013-14.pdf

[74] Pakistan Telecommunication Authority. Paradigm Technologies Broadband Subscribers Survey: Estimating Broadband End-Users their Experience of Service its Performance. Pakistan Telecommunication Authority (2010). Available online at: https://www.pta.gov.pk/media/bb_sub_sur_report_10.pdf

[75] Hosen I Al Mamun F Mamun MA. The role of sociodemographics, behavioral factors, and internet use behaviors in students' psychological health amid COVID-19 pandemic in Bangladesh. Health Sci Rep. (2021) 4:e398. 10.1002/hsr2.398 34622029 PMC8485611

[76] Hosen I Al Mamun F Sikder MT Abbasi AZ Zou L Guo T. Prevalence and associated factors of problematic smartphone use during the COVID-19 pandemic: a Bangladeshi study. Risk Manag Healthc Policy. (2021) 14:3797–805. 10.2147/RMHP.S325126 34548828 PMC8448157

[77] Karachi's rural population increases by over 275pc in census - Pakistan - DAWN.COM n.d. Available online at:https://www.dawn.com/news/1354776 (accessed February 18, 2022).

[78] Hasan A. Emerging urbanisation trends: The case of Karachi (2016).

[79] Hasan A Mohib M. The case of Karachi, Pakistan. Urban Slums Rep (1998). p.1–32.

